# 糖皮质激素治疗阵发性睡眠性血红蛋白尿症的局限性与挑战

**DOI:** 10.3760/cma.j.cn121090-20241213-00568

**Published:** 2025-03

**Authors:** 冰 韩

**Affiliations:** 中国医学科学院北京协和医院血液科，北京 100730 Department of Hematology, Peking Union Medical College Hospital, Chinese Academy of Medical Sciences and Peking Union Medical College, Beijing 100730, China

**Keywords:** 阵发性睡眠性血红蛋白尿症, 糖皮质激素, Paroxysmal nocturnal hemoglobinuria, Glucocorticoids

## Abstract

阵发性睡眠性血红蛋白尿症（Paroxysmal nocturnal hemoglobinuria, PNH）是一种罕见的获得性造血干细胞疾病，多起病于青壮年，特征是骨髓衰竭、持续的血管内溶血和血栓形成，均可引起严重的终末器官损伤，增加早期死亡的风险，造成患者严重的疾病负担。在我国，针对PNH患者的治疗尚未与国际同步，糖皮质激素仍然是临床常规用药。然而，糖皮质激素对于PNH溶血的控制机制并不明确，缺少支持其使用的循证医学数据和临床获益经验，且长期使用糖皮质激素显著增加患者不良反应的发生风险。鉴于PNH需要终身管理，长期糖皮质激素治疗将严重损害患者健康，国内外指南/专家共识均不建议应用糖皮质激素。本文针对糖皮质激素治疗PNH患者的局限与挑战进行论述，以促进中国PNH治疗策略的更迭。

## Abstract (en)

Paroxysmal nocturnal hemoglobinuria（PNH）is a rare acquired hematopoietic stem cell disease that mainly occurs in young adults and is characterized by bone marrow failure, persistent intravascular hemolysis and thrombosis, all of which can cause severe end-organ damage, increase the risk of early death, and cause a severe disease burden in patients. In China, based on the historic reasons, glucocorticoids are still routinely used in many places. However, the effects of glucocorticoids on PNH hemolysis are uncertain. Evidence-based medical data and clinical benefits for glucocorticoid on PNH are missing, but the long-term use of glucocorticoids significantly increases the risk of adverse reactions in patients. Since PNH needs a lifelong follow-up and management, long-term glucocorticoid therapy will unavoidably seriously damage the health of patients. Therefore, glucocorticoids are not recommended for the treatment of PNH, either from domestic or overseas guidelines, or expert consensus. In this article, the limitations and challenges of glucocorticoids in the treatment of PNH were expounded upon, in order to encourage more effective and safe strategies to be accepted in China.

阵发性睡眠性血红蛋白尿（Paroxysmal nocturnal hemoglobinuria, PNH）是一种罕见的后天获得性造血干细胞克隆性疾病[1]。PNH全球年发病率为（1～2）/100万人，患病率为（10～20）/100万人，而我国PNH年发病率至今没有非常精确的统计，牡丹江地区曾统计为2.85/100万人，可能较欧美国家常见[1]–[3]。PNH的发病机制是X染色体上的磷脂酰肌醇聚糖锚生物合成A类基因（Phosphatidylinositol glycan anchor biosynthesis class A, PIG-A）突变，引起糖基化磷脂酰肌醇（Glycosylphophatidylinositol, GPI）锚的合成缺陷，导致造血干细胞及向下分化的血细胞表面上糖基磷脂锚定蛋白（GPI-AP）的缺失，使细胞抵抗补体攻击的能力减弱，从而导致细胞被破坏而发生溶血。其中，两种必需的补体调节蛋白CD55和CD59的缺乏导致PNH红细胞易发生补体介导的溶血[3]–[4]。

PNH的发病年龄在各年龄组均有报道，20～40岁占77%，以血管内溶血、血栓形成和骨髓衰竭等为特征[5]。该病起病隐匿，病程进展缓慢，常累及全身多系统，出现较为复杂的临床表现，患者的生活质量、身体功能和工作效率下降，严重者可危及生命。尽管PNH是一种良性疾病，但在传统治疗模式下，患者5年总生存率仅为66.8%[6]。

自2007年补体抑制剂获得FDA批准以来，国外已建立以补体抑制剂为主的标准治疗模式。在我国，约91.3%的PNH患者仍然接受对症支持治疗为主的传统治疗模式，包括糖皮质激素、铁剂、抗凝药物、雄激素、免疫抑制剂等，其中超过80%的患者接受了糖皮质激素治疗[7]。然而，临床实践表明，糖皮质激素疗效欠佳，长期使用给患者带来诸多严重不良反应。2023年我国开展的糖皮质激素治疗PNH的前瞻性临床试验结果，均不推荐长期糖皮质激素维持治疗PNH[8]–[9]。此外，一项针对我国PNH患者的调研报告发现，53.8%的患者出现中/重度疲劳或贫血，且超过40%的患者认为传统的治疗方案并未有效改善症状[10]。基于此，本文将主要围绕使用糖皮质激素治疗PNH的利害关系做一阐述。

一、应用糖皮质激素治疗PNH的可能机制

PNH的核心发病机制是机体内末端补体系统呈持续活化状态而不被抑制，血管内破坏红细胞而引发的多系统损伤[11]。糖皮质激素具有广泛的抗炎和免疫抑制作用，可通过反式激活抗炎蛋白［如糖皮质激素诱导的亮氨酸拉链（GILZ）］和反式抑制促炎基因（如编码促炎细胞因子的基因），发挥抗炎和免疫抑制作用，使其在慢性炎症性疾病、过敏和自身免疫性疾病中有效，但对于补体系统的抑制作用尚不明确[12]。既往使用糖皮质激素的原因，推测可能是考虑到在PNH急性血管内溶血时，糖皮质激素通过抗炎作用增加机体应激能力及清除游离血红蛋白，减少血红蛋白引发的毒性过程，降低急性溶血时不良事件的发生率[9]。但本质上，糖皮质激素并不能解决PNH的末端补体激活这一根本病因，并不能避免补体介导的溶血发生。因此，无论是短期还是长期，糖皮质激素都无法控制PNH的溶血。

二、糖皮质激素治疗PNH的局限性

PNH患者采用糖皮质激素起始治疗后，根据病情和血液学指标逐步调整剂量，属于经验性用药，缺乏临床证据。以糖皮质激素为代表的传统治疗手段对临床上大多数难治和复发患者无效。糖皮质激素对少部分初诊患者可能减轻溶血发作、稳定病情。但持续治疗后，患者多会出现激素无效或依赖。许多患者长期使用糖皮质激素出现了各种严重的不良反应[13]。

目前，国际上没有证据支持长期使用糖皮质激素治疗PNH获益。一项研究纳入了在韩国PNH登记中心注册的97例PNH患者，入组后被分为激素治疗组和非激素治疗组，随访至少6个月，评估激素治疗在PNH患者慢性溶血中的作用。结果显示激素治疗PNH无法控制溶血，仍有高达85.7%的PNH患者经历血管内溶血，伴发腹痛、呼吸困难，且需要红细胞输注的比例更高[14]。我国杨慧等[9]开展的一项糖皮质激素维持治疗PNH的研究纳入了26例疾病高活动状态的患者，前瞻性自身对照比较发现，泼尼松1.0 mg·kg^−1^·d^−1^治疗后1、3、6个月无患者达到溶血控制（LDH<1.5×正常上限）。血清LDH、血红蛋白、间接胆红素水平、外周血网织红细胞比例、D-二聚体水平、肾小球滤过率治疗前后未见改善。另一项纳入40例PNH高活动状态患者的糖皮质激素维持治疗（泼尼松1.0 mg·kg^−1^·d^−1^）的类似研究发现，泼尼松最初可能改善贫血，但研究者认为贫血的改善可能是由于PNH本身发作后的自我缓解或输血[8]。德国一项研究报道了1例56岁的男性PNH病例接受了大约15年的泼尼松治疗，患者出现频繁的溶血发作以及血栓和肾功能恶化，包括长期糖皮质激素治疗的并发症，后续该患者调整为依库珠单抗治疗后迅速改善了血红蛋白和LDH水平，溶血发作完全停止，而且表现出良好的耐受性[15]。以上研究均证实糖皮质激素在维持治疗PNH过程中不能有效控制溶血，无法改善贫血、血栓情况及肾功能。

尽管上述报道都显示糖皮质激素对于PNH的治疗是无效的，但由于其价格便宜、方便使用，部分患者治疗期望值低，导致其在我国仍然被广泛应用。事实上，糖皮质激素维持使用的临床证据不足、获益不明确，而且会引起多种不良反应，误用滥用甚至会加速PNH患者病情的恶化。

三、糖皮质激素治疗PNH的安全性挑战

糖皮质激素是各种免疫炎症相关疾病中减少炎症和免疫激活的标准疗法，但糖皮质激素的潜在不良反应发生率很高，高达90%的服用糖皮质激素超过60 d的患者，可能出现不同的不良反应，即使在服用低剂量（≤7.5 mg/d）的患者中，也可能出现这些不良反应[16]。糖皮质激素相关的不良反应可涉及肌肉骨骼、胃肠、心血管、内分泌、神经精神、皮肤、眼部和免疫系统。我国一项单中心观察性研究（2005–2015年）发现81例经典型PNH患者的糖皮质激素平均治疗时长为7.5个月，最常见的不良事件是医源性库欣综合征、感染、骨质疏松症和高血糖[17]。

糖皮质激素具有强大的免疫抑制作用，会抑制多种免疫细胞的功能，从而降低机体的防御能力，导致细菌、病毒、真菌、寄生虫等致病微生物的活动性增强，繁殖加快，引起感染扩散、炎症反复等。一项英国风湿研究队列研究显示，长期应用糖皮质激素的患者感染风险增加1.83倍，即使小剂量激素（5 mg/d）应用3个月，严重感染的住院风险仍增加30%[18]。对于PNH患者这类特殊的人群，任何一次感染都可能导致病情反复和恶化，临床应警惕。一项纳入7个大样本量队列研究的荟萃分析结果显示口服泼尼松2.5～7.5 mg/d患者发生骨折的可能性增加57%，发生髋部骨折的可能性增加125%[19]–[20]。小剂量糖皮质激素维持治疗狼疮性肾炎12个月后，骨密度降低，骨质疏松发生率为20%[21]。近年来相关研究证实即使生理剂量的糖皮质激素也可引起骨量减少，所以在应用糖皮质激素治疗疾病时，不管剂量高低，均应关注其可能引起的骨量减少或骨质疏松，及增加的骨折风险[22]。

使用较大剂量的糖皮质激素可能是静脉血栓栓塞（Venous thromboembolism, VTE）的另一风险因素，而长期小剂量治疗则是动脉血栓形成的风险因素[23]。糖皮质激素治疗与动静脉血栓发生风险增加有关[24]–[25]。糖皮质激素可增加凝血和抑制纤维蛋白溶解，糖皮质激素使用者患VTE的风险增加。丹麦一项大规模的、基于人群的病例对照研究（VTE组和对照组各38 765例）发现使用全身性激素初期（即处方时间与调查时间间隔≤90 d）发生VTE事件的可能性是未使用激素者的2.31倍，且随糖皮质激素累积暴露剂量的增加而增加[26]。使用糖皮质激素治疗期间VTE复发的风险是未使用激素者的2.72倍[27]。糖皮质激素增加VTE风险还与合并的基础疾病有关[28]。可见，在有/无诱因的作用下,使用糖皮质激素均可增加VTE风险。基于英国心脑血管疾病队列的巢式病例对照研究，发现曾经使用过口服糖皮质激素与心脑血管病发生的可能性存在显著关联（*OR*＝1.25, 95% *CI* 1.21～1.29）；且使用激素初期的影响最大，发生心力衰竭和缺血性心脏病可能性分别是未使用激素者的2.66倍和1.20倍[29]。

美国早在1953年就提出激素对于PNH的治疗没有价值[30]。随着我们对糖皮质激素治疗PNH疗效和潜在不良反应认识的不断深入，近20年来国外专家反复提及糖皮质激素治疗效果有限，尚没有循证证据表明长期使用对PNH有益，并且长期使用的不良反应显著，糖皮质激素不应被视为PNH的一线治疗。

四、国内外对于糖皮质激素治疗PNH的见解

目前国外专家已摒弃糖皮质激素作为PNH的治疗选择，建议不使用或仅在合并其他需要用糖皮质激素情况下使用。

巴西PNH诊断和治疗共识[3]认为糖皮质激素虽然被广泛使用，但激素是一种经验疗法，没有随机对照试验研究数据，也没有明确的疗效。他们指出，仅在特定情况下，短期（几天）糖皮质激素治疗可用于减轻溶血危机的严重程度和缩短持续时间。土耳其PNH教育和研究小组的指南同样提出只在溶血发作时考虑短期使用激素，不建议长期使用[31]。比利时专家小组的建议则指出长期糖皮质激素治疗的不良反应和较差的疗效限制了其使用[32]。无论是慢性溶血还是急性溶血加重，使用糖皮质激素治疗都存在争议，国际PNH兴趣小组不主张在任何情况下使用糖皮质激素治疗PNH，长期使用可能产生严重危害[33]。

《PNH诊断与治疗中国指南（2024年版）》及《PNH克隆筛查及补体抑制剂治疗监测中国专家共识（2024年版）》均提出PNH的一线治疗方案为补体抑制剂，可选择依库珠单抗、可伐利单抗和B因子抑制剂[1],[4]。建议当急性溶血发作时，无可及补体抑制剂时可给予糖皮质激素（如泼尼松0.25～1 mg·kg^−1^·d^−1^，为避免长期应用的不良反应，应酌情短周期使用），不建议糖皮质激素用于维持治疗[1]。实际上，即使短期使用激素控制溶血发作，也缺乏循证医学证据，更多的是经验性选择。

五、补体抑制剂是PNH的标准治疗手段

随着补体领域的探索，补体抑制剂成为治疗PNH的有效药物，PNH患者的生存率已经接近性别和年龄匹配的对照组，PNH的一线治疗选择也转变为补体靶向治疗。目前在我国获批用于治疗PNH的有依库珠单抗、可伐利单抗、伊普可泮3种补体抑制剂[4]。首个靶向末端补体C5抑制剂依库珠单抗，在临床试验中被证明对PNH患者具有高度有效性和良好的耐受性，并于2007年在美国和欧洲获批上市，该药已于2022年在中国上市。长期队列研究显示，与接受依库珠单抗治疗前的传统治疗模式相比，接受依库珠单抗治疗显著降低了死亡风险（*HR*＝0.21, 95% *CI* 0.05～0.88），使患者5年生存率由66.8%提升至95.5%，并减轻溶血程度、降低血栓风险，是目前临床数据及经验最丰富的补体药物[6]。长效C5抑制剂可伐利单抗与依库珠单抗相比，两组在血红蛋白稳定和疲劳评分的改善方面显示出了相似的结果，安全性相当[34]。近端补体抑制剂伊普可泮对于C5抑制剂治疗后存在血管外溶血的经典型PNH成年患者，可进一步改善患者血红蛋白水平[35]。

即使在无法使用补体抑制剂或造血干细胞移植的情况下，对症支持，包括输血、抗凝及其他支持治疗，也能改善短期的症状，待患者条件允许后，再进行其他有效的治疗。如果滥用糖皮质激素，不仅无效，还可能造成糖皮质激素相关并发症，使患者丧失其他治疗的机会。

六、总结

糖皮质激素的对症治疗，短时间内很难改善PNH患者的急性溶血症状，长期治疗更无获益。在维持治疗过程中，不能控制溶血，也不能改善贫血、血栓形成情况及肾功能，并且随着治疗时间的延长会导致各种严重的不良反应。PNH的治疗目标，除短期内快速控制溶血外，更需要关注患者的长期预后。随着补体抑制剂在中国获批上市并且可及性不断提升、各种PNH新药临床试验的广泛开展以及异基因造血干细胞移植技术的不断进步，PNH患者的治疗将会有更多选择。
